# Supplementary figures and images for: A neural network account of memory replay and knowledge consolidation
Source: Cereb Cortex. 2022 Feb 25;33(1):83–95. doi: 10.1093/cercor/bhac054 (PMC9758580; doi:10.1093/cercor/bhac054)

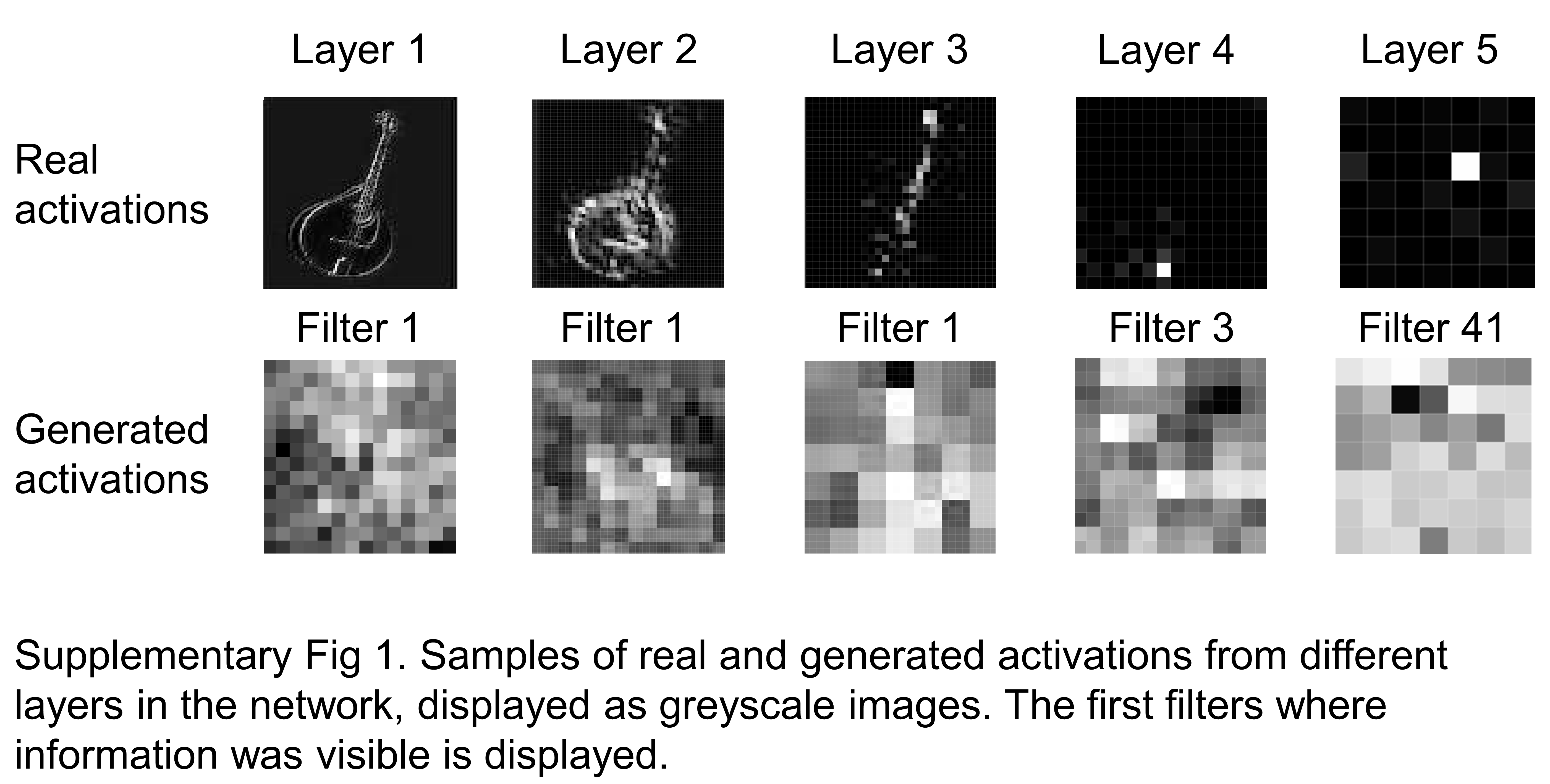

Supplement: Supplementary_Fig_1_bhac054 [file supplementary_fig_1_bhac054.zip › Supplementary_Fig_1_bhac054.tif]

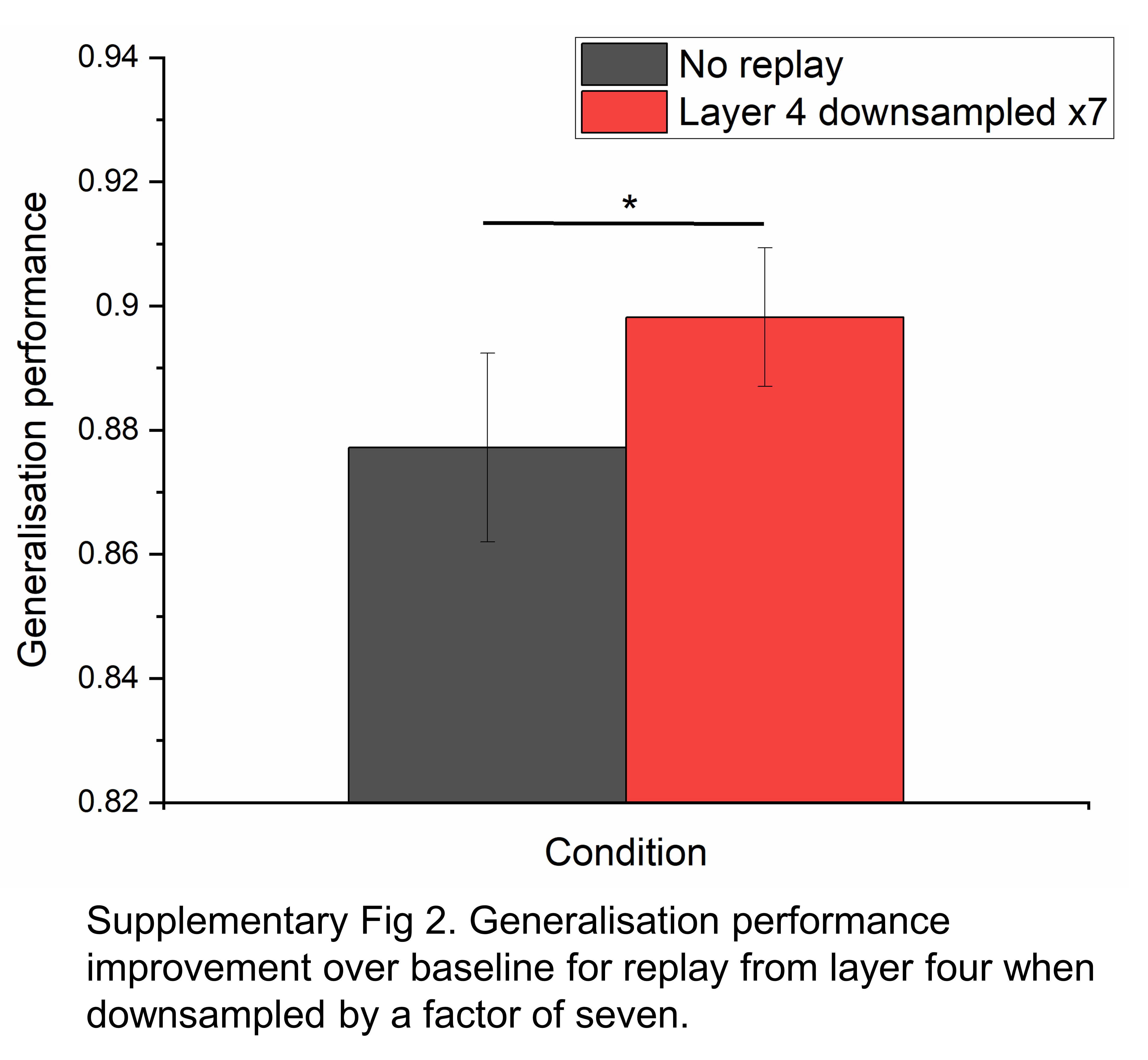

Supplement: Supplementary_Fig_2_bhac054 [file supplementary_fig_2_bhac054.zip › Supplementary_Fig_2_bhac054.tif]
