# Supplementary material for: A neural network account of memory replay and knowledge consolidation
Source: Cereb Cortex. 2022 Feb 25;33(1):83–95. doi: 10.1093/cercor/bhac054 (PMC9758580; doi:10.1093/cercor/bhac054)
Supplement: Supplementary_table_1_bhac054 [file supplementary_table_1_bhac054.docx]

Supplementary table 1: List of ImageNet classes by model

| Model 1 | n12360108 begonia |
| --- | --- |
|  | n02822579 bedstead bedframe |
|  | n02427724 waterbuck |
|  | n03098688 control room |
|  | n02944075 camisole |
|  | n01603600 waxwing |
|  | n03196598 digital display alphanumeric display |
|  | n02848216 blade |
|  | n07712856 tortilla chip |
|  | n03592669 jalousie |
| Model 2 | n11853356 Christmas cactus Schlumbergera buckleyi Schlumbergera baridgesii |
|  | n04177820 settle settee |
|  | n03904183 pedestrian crossing zebra crossing |
|  | n04355511 sundress |
|  | n03487444 hand lotion |
|  | n12899752 angel's trumpet Brugmansia suaveolens Datura suaveolens |
|  | n12655869 raspberry raspberry bush |
|  | n12948053 common European dogwood red dogwood blood-twig pedwood Cornus sanguinea |
|  | n02869737 bongo bongo drum |
|  | n02415253 Dall sheep Dall's sheep white sheep Ovis montana dalli |
| Model 3 | n03375575 foil |
|  | n03082807 compressor |
|  | n03262932 easy chair lounge chair overstuffed chair |
|  | n02047614 puffin |
|  | n03317788 faience |
|  | n09475044 wasp's nest wasps' nest hornet's nest hornets' nest |
|  | n11784497 jack-in-the-pulpit Indian turnip wake-robin Arisaema triphyllum Arisaema atrorubens |
|  | n03941231 pinata |
|  | n02813399 bay window bow window |
|  | n04544325 wainscoting wainscotting |
| Model 4 | n03993053 potty seat potty chair |
|  | n04082886 reticle reticule graticule |
|  | n03421324 garter belt suspender belt |
|  | n03766044 miller milling machine |
|  | n03505504 headscarf |
|  | n12384839 love-in-a-mist running pop wild water lemon Passiflora foetida |
|  | n03619793 kitbag kit bag |
|  | n07600696 candied apple candy apple taffy apple caramel apple toffee apple |
|  | n02068974 dolphin |
|  | n03237992 dressing gown robe-de-chambre lounging robe |
| Model 5 | n02918964 bumper car Dodgem |
|  | n02392824 white rhinoceros Ceratotherium simum Diceros simus |
|  | n01806364 blue peafowl Pavo cristatus |
|  | n02956699 capitol |
|  | n04290079 spun yarn |
|  | n08596076 littoral litoral littoral zone sands |
|  | n02887970 bracelet bangle |
|  | n10635788 sphinx |
|  | n07901457 muscat muscatel muscadel muscadelle |
|  | n07870167 lasagna lasagne |
| Model 6 | n04324387 stockroom stock room |
|  | n04591517 wind turbine |
|  | n02988486 CD-R compact disc recordable CD-WO compact disc write-once |
|  | n04568069 weathervane weather vane vane wind vane |
|  | n04514241 uplift |
|  | n03207835 dishtowel dish towel tea towel |
|  | n13206817 maidenhair maidenhair fern |
|  | n03307792 external drive |
|  | n12666965 cape jasmine cape jessamine Gardenia jasminoides Gardenia augusta |
|  | n12950126 valerian |
| Model 7 | n03986355 portfolio |
|  | n11848479 night-blooming cereus |
|  | n04439712 tinfoil tin foil |
|  | n03160740 damask |
|  | n01612122 sparrow hawk American kestrel kestrel Falco sparverius |
|  | n09206896 arroyo |
|  | n12392549 stinging nettle Urtica dioica |
|  | n02343772 gerbil gerbille |
|  | n07875436 risotto Italian rice |
|  | n02060133 fulmar fulmar petrel Fulmarus glacialis |
| Model 8 | n03655072 legging leging leg covering |
|  | n10738111 unicyclist |
|  | n09270735 dune sand dune |
|  | n03409393 gable gable end gable wall |
|  | n02331046 rat |
|  | n03452267 gramophone acoustic gramophone |
|  | n10105733 forward |
|  | n07911677 cocktail |
|  | n03797182 muffler |
|  | n01563128 warbler |
| Model 9 | n04197110 shipwreck |
|  | n10470779 priest |
|  | n02769290 backhoe |
|  | n03478756 hall |
|  | n04519153 valve |
|  | n04289027 sprinkler |
|  | n02782778 ballpark park |
|  | n03558404 ice skate |
|  | n04138261 satin |
|  | n02700064 alternator |
| Model 10 | n03524150 hockey stick |
|  | n03716966 mandolin |
|  | n02962200 carburetor carburettor |
|  | n03237340 dresser |
|  | n04004210 printed circuit |
|  | n02917377 bullhorn loud hailer loud-hailer |
|  | n07879953 tempura |
|  | n04087826 ribbing |
|  | n02404432 longhorn Texas longhorn |
|  | n07830593 hot sauce |
