# Supplementary material for: A neural network account of memory replay and knowledge consolidation
Source: Cereb Cortex. 2022 Feb 25;33(1):83–95. doi: 10.1093/cercor/bhac054 (PMC9758580; doi:10.1093/cercor/bhac054)
Supplement: Supplementary_table_2_bhac054 [file supplementary_table_2_bhac054.docx]

| Model 1 (1) | n12360108 begonia |
| --- | --- |
| Model 2 (1) | n11853356 Christmas cactus Schlumbergera buckleyi Schlumbergera baridgesii |
| Model 2 (6) | n12899752 angel's trumpet Brugmansia suaveolens Datura suaveolens |
| Model 2 (8) | n12948053 common European dogwood red dogwood blood-twig pedwood Cornus sanguinea |
| Model 4 (6) | n12384839 love-in-a-mist running pop wild water lemon Passiflora foetida |
| Model 6 (7) | n13206817 maidenhair maidenhair fern |
| Model 6 (9) | n12666965 cape jasmine cape jessamine Gardenia jasminoides Gardenia augusta |
| Model 6 (10) | n12950126 valerian |
| Model 7 (2) | n11848479 night-blooming cereus |
| Model 7 (7) | n12392549 stinging nettle Urtica dioica |

Supplementary table 2: Conceptually similar classes (plants)
